# Social Workers in Primary Care Increase Access to Palliative Care

**DOI:** 10.1093/geroni/igab046.3047

**Published:** 2021-12-17

**Authors:** Portia Cornell, Christopher Halladay, Pedro Gozalo, Caitlin Celardo, James Rudolph, Anna-Rae Montano

**Affiliations:** 1 Providence VA Medical Center, Providence, Rhode Island, United States; 2 Brown University, Providence, Rhode Island, United States; 3 Northport VA Medical Center, Northport VA Medical Center, New York, United States

## Abstract

Clinical trials show that palliative care improves patient experiences and reduces costs, and use of palliative care and hospice care have been increasing over the past three decades. In the Veterans Administration health care system (VA), Veterans may receive palliative care concurrently with other treatments. However, many barriers exist to the use of palliative care, such as patients’ misperceptions. Social workers in primary care teams may increase use of this valuable service by establishing trust between patient and care team, educating patients and caregivers, and coordinating services. Leveraging a national social-work-staffing program as a natural experiment, we evaluated the effect of hiring one or more social workers to the primary-care team on use of palliative or hospice care among Veterans with a recent hospital stay. Our data included 91,675 episodes of care between 2016 and 2018. 1.45 percent of episodes were followed by use of palliative care or hospice within 30 days. The addition of one or more social workers through the staffing program was associated with an increase of 0.53 percentage points (p<0.001) in the probability of any palliative or hospice care, i.e., a more than 30% increase relative to the mean. Policy makers and health system leaders who seek to improve patient experience and reduce costs through increased access to palliative and hospice care could consider social work staffing as a policy tool to achieve those aims.

